# Innovative strategies for valorization of byproducts from soybean industry: A review on status, challenges, and sustainable approaches towards zero-waste processing systems

**DOI:** 10.1016/j.heliyon.2025.e42118

**Published:** 2025-01-25

**Authors:** Ahasanul Karim, Emmanuel Freddy Osse, Seddik Khalloufi

**Affiliations:** a Department of Soils and Agri-Food Engineering, Université Laval, Quebec, QC, G1V 0A6, Canada; b Institute of Nutrition and Functional Foods (INAF), Université Laval, Quebec, QC, G1V 0A6, Canada

**Keywords:** Soybean industry, Valorization of byproducts, Value-added products, Zero-waste processing system, Sustainable industry

## Abstract

The agro-food supply chain generates significant quantities of waste and byproducts globally, influenced by regional socioeconomic conditions, policy frameworks, and environmental concerns. The soybean industry generates various byproducts during the production processes of oil, soy milk, tofu, soy yogurt, edamame, miso, tempeh, natto, and soy sauce, presenting both challenges and opportunities for sustainable valorization. The review aims to outline the composition, status, and potential applications of key byproducts within the soybean industry including soy okara, soy whey, soy hull, soy meal, and lecithin, elucidating innovative strategies for their comprehensive valorization. The goal is to establish a sustainable zero-waste processing system by effectively utilizing these byproducts. This review explores emerging biotechnological advancements and eco-friendly processes aimed at maximizing resource recovery through the valorization of these soy byproducts. Various commercially viable products derived from repurposing the carbohydrate and protein fractions of diverse soy byproducts are highlighted. Additionally, a cutting-edge framework is proposed, advocating for the establishment of a zero-waste system within the soybean processing sector, emphasizing integrated biorefinery technologies, circular economy strategies, and sustainability principles. The framework proposed encompasses maximizing okara utilization, extracting value-added products, and implementing a closed-loop byproduct management approach within collaborative supply chains. Despite promising prospects, challenges such as anti-nutrients, viscosity and solubility of soy powder, and environmental impact must be addressed. This study could inspire further research into innovative technologies for the comprehensive and integrated valorization of soy byproducts, aiming to mitigate food waste and enhance the sustainability of the soybean industry.

## Highlights

• Review of status and sustainable valorization strategies for soybean byproducts.
• Innovative advancements for maximizing resource recovery from soy byproducts.
• Proposed framework for achieving zero-waste processing in the soybean industry.
• Challenges and valorization strategies for sustainable management of soy byproducts.

## Graphical abstract

**Figure:**
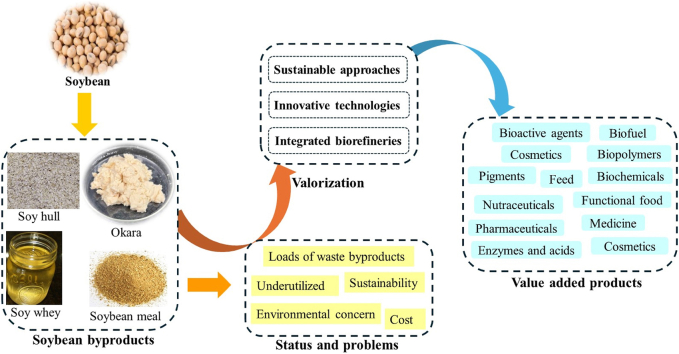

## 1 Introduction

The common industrial practice of disposing of food processing byproducts leads to economic losses and socio-environmental challenges. This is why the search for other uses for these food streams and their valorization has garnered great attention worldwide in recent years [1]. This is particularly relevant in the context of soybean industries, which produce substantial quantities of waste annually, posing ecological disadvantages [[1], [2], [3]]. The surge in consumer interest in Soy (*Glycine max*) has resulted in a significant increase in soybean production over the past few decades, with projections reaching 371 million tonnes by 2030 [4,5]. According to U.S. Department of Agriculture data, global soybean production in 2020 totaled approximately 336 million tons [6]. Currently, Brazil is the largest producer of soybeans with 133 million tons, followed by the United States with 113 million tons, Argentina with 48 million tons, China with 20 million tons, India with 10 million tons, and other countries contributing 12 million tons [7].

Consequently, the soy food industry is experiencing remarkable growth, with projections indicating a substantial increase in the market size of soy food products. According to Singh and Krishnaswamy [7], the market is expected to expand from USD 45 billion in 2021 to USD 61 billion by 2027. Soy-based food products, including soy milk, tofu, soy yogurt, edamame, miso, tempeh, natto, and soy sauce, have gained significant popularity due to their favorable nutritional profiles characterized by low cholesterol levels and high protein content. Additionally, a wide variety of food items such as bean sprouts, baked soy goods, fermented soy products and beverages, sweets, cookies, soy-nut butter, quick milk drinks, textured soy protein, extruded soy products, plant protein concentrates, and diet foods are readily available for consumption [1,7]. Two significant byproducts, okara and soy whey, are generated during the liquid extraction process of soy food processing. These byproducts, rich in nutrients and medicinal attributes, find applications in the production of diverse food items, pharmaceuticals, and industrial goods. Soybeans and their byproducts also yield a range of medicinal chemicals, including isoflavones, saponins, lecithin, phytic acids, glycine, bioactive peptides, and protein supplements [8,9].

**Fig. 1:**
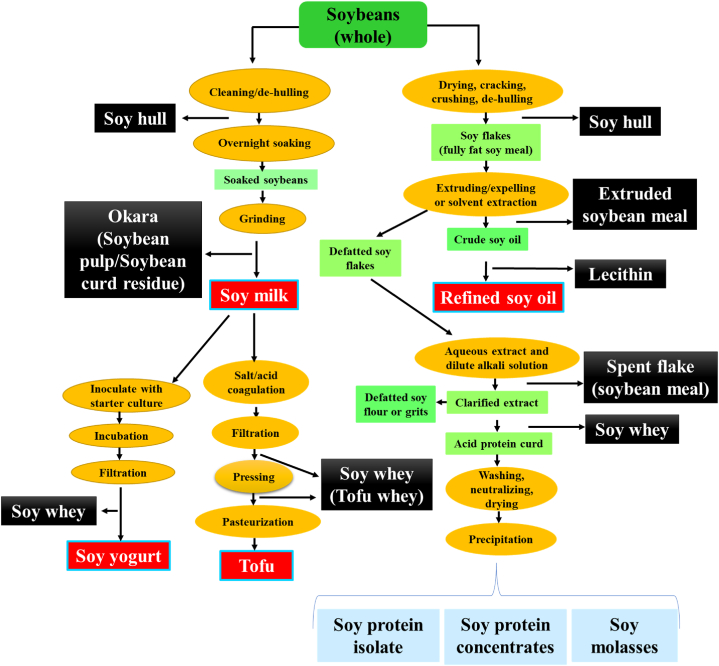
Process flow diagram for production of soy-based foods and their byproducts.

Additionally, it is imperative to emphasize that the soy oil industry is presently assessed at $20 billion, with forecasts indicating a significant expansion, poised to reach $33 billion by 2031 [7]. The rising prominence of soybean oil can be ascribed to its extensive application across diverse industries, spanning food, nutraceuticals, biodiesel, polymers, resins, pesticides, and animal feeds [10,11]. In addition to their primary products, soybean oil industries generate substantial quantities of valuable byproducts and waste materials, including soy meal, soy hull, lecithin, expelled soy meal cake, and de-fatted soy flakes. It is worthy to mention that approximately 5 kg of soybeans is used to produce 1 L of soy oil, with the remaining 75–80 % generated as a byproduct [12]. As depicted in Fig. 1, many of these byproducts are strategically repurposed in the manufacturing of animal feed, soy protein concentrate (SPC), and soy protein isolates (SPI). Furthermore, a portion of these residual substances finds purpose in the production of biodiesel, ethanol, cosmetics, bioplastics, emulsifiers, adhesives, paints, disinfectants, and various other industrial applications [13].

Despite significant efforts in valorizing soy byproducts, many methods still generate considerable waste, underscoring the urgency of aligning with circular economy principles [7,13]. Transforming these byproducts into valuable products is essential for reducing waste and improving resource recovery. However, current valorization technologies lack universal applicability, scalability, and cost-effectiveness, particularly for byproducts like soy whey, okara, soy hull, and lecithin. Integrating various valorization processes—such as fermentation, enzymatic treatment, and extraction—into cohesive, circular economy models remains a critical challenge [14,15]. Additionally, high operational costs, especially for small-scale operations, and environmental concerns related to energy and water usage hinder the broader adoption of these processes [16,17].

The terms "food waste" and "byproducts" are often used interchangeably, though they denote distinct concepts [16]. European legislation defines “food waste” as discarded residues with high organic content from food production, while “food byproducts” refer to materials that, despite being categorized as waste, can serve as valuable substrates for recovering functional compounds. This shift emphasizes the potential of these byproducts as resources for creating marketable products and supports more sustainable practices in food processing [18]. In this study, “food waste” refers to all food fractions removed from the supply chain, whereas “byproduct” specifically denotes waste materials that are valorized or have potential for valorization.

While promising technologies exist for valorizing soy byproducts, they remain largely at the pilot scale, with limited success in scaling up to industrial levels. This highlights the urgent need for innovative valorization techniques that minimize waste generation and enable the full reuse of byproducts in the soybean industry [19,20]. Overcoming challenges such as allergens, environmental impact, and cost factors is crucial for advancing sustainable practices and achieving zero-waste systems. This review critically examines the current state of soybean byproduct utilization, highlighting potential applications, emerging strategies especial for food and feed applications, and the limitations that impede progress toward a circular bioeconomy in the sector. This review critically assesses the current state of soybean byproduct utilization, highlighting potential applications. It emphasizes emerging strategies, particularly in the food and feed sectors, and discusses the challenges that hinder progress toward a circular bioeconomy, aiming to achieve a zero-waste objective in the industry.

## 2 Byproducts from soy industry and their composition

### 2.1 Byproducts derived from soy milk extraction and tofu production

#### 2.1.1 Soy okara

Soybean curd residue, commonly known as okara, is a byproduct generated during the processing of soybeans (Fig. 1). The increased consumption of soy-based products has resulted in significant quantities of okara, which is the ground soy residue remaining after filtration of the water-soluble fraction during soy milk and tofu production [1,21]. Its primary constituents consist of damaged cotyledon cells and the outer layer of soybeans, and it is rich in nutrients, retaining nearly all the nutrients found in soybeans. Despite being a byproduct, okara maintains a nutrient-rich composition, comprising approximately 40–60 % fiber, 20–30 % protein, 10–20 % fat on a dry basis, as well as minerals and phytochemicals [1,20,22,23]. Table 1 presents the approximate chemical composition of each component, detailing their content of lipids, proteins, carbohydrates, and minerals. These values may fluctuate depending on factors such as variety, geographical origin, and climatic conditions. Fresh okara contains around 80 % water, 3.5–4.0 % protein, and a majority of insoluble components, while containing fraction of soluble constituents due to its high moisture content [24]. Although soy okara is rich in protein and has a beneficial amino acid profile, it also contains anti-nutritional factors such as trypsin inhibitors, goitrogenic factors (e.g., soy isoflavones), saponins, phytoestrogens, and mineral-binding substances. These compounds can hinder nutrient digestion and absorption, making it less suitable for direct human consumption [25].

**Table 1:** Composition of soybean and various byproducts from soy industry. The chemical composition of these components, including their lipid, protein, carbohydrate, and mineral content, can vary based on factors such as variety, geographic location, and weather conditions.

Byproducts	Moisture	Protein	Fiber	Fat	Carbohydrates	Ash	Total solids	Reference
Soybean	8.20–10.00	39.50–50.00	3.20–5.50	18.20–21.00	30.00–35.00	3.61–5.40	88.00–92.00	[7,32,33]
Okara (wet)	68.03	8.08	5.00	6.02	12.01	1.00	32.05	[34]
Okara (dried)	1.00	34.15	33.00	12.60	48.90	2.05	95.04	[34]
Soy whey (Tofu)	80.00–90.00	0.1–0.8	–	0.4–1	1	0.40	–	[9,35]
Soy hull	7.00–10.00	11.40–12.80	34.80–39.01	2.00–4.00	30.00–40.00	4.10–5.30	90.00–93.00	[[36], [37], [38]]
Soy meal	11.02	33.04–56.27	4.30–7.20	0.50–3.17	4.66–7.00	5.60–7.20	–	(Sharma et al., 2014) [39]
Lecithin	0–1.00	0–1.00	–	80.00–95.00	5.00–6.00	–	–	[40,41]

#### 2.1.2 Soy whey

Soy whey is a byproduct generated during the processing of soy yogurt, tofu, SPI, SPC, and other fermented soy products. Its production primarily involves two sources: tofu production and SPI production (Fig. 1). In tofu production, soaked soybeans are crushed with water to obtain soy milk, which is then heated and coagulated using calcium/magnesium salt or an acid, resulting in tofu and soy whey [26]. SPI production entails defatting soy flakes, extracting proteins, washing, neutralization, and drying [27]. Soy whey contains carbohydrates, nitrogen compounds, and minerals that support microbial growth. It has a high biological oxygen demand (BOD) ranging from 8000 to 9800 mg/L and a chemical oxygen demand (COD) ranging from 17000 to 26000 mg/L [28,29]. Soy whey from tofu manufacturing retains significant nutrients found in soy milk. It typically contains approximately 1 % carbohydrates (8.50 g/L), primarily stachyose and sucrose, with smaller amounts of fructose, glucose, and raffinose. Protein concentration ranges from 0.1 % to 0.8 % (1.33–8.20 g/L), while lipid concentration ranges from 0.4 % to 1.0 % (3.9–10.0 g/L). Mineral content is about 0.4 %, with concentrations ranging from 3.9 to 4.6 g/L [9,30]. Conversely, there is limited documentation on the constituents of SPI-derived soy whey compared to tofu-derived soy whey. SPI-derived soy whey primarily comprises carbohydrates (9.50 g/L), proteins (0.3–3.00 g/L), and minerals (1.93 g/L) [9,31]. Soy whey composition varies based on source, environmental conditions, and processing methods, with tofu-derived soy whey retaining more nutrients from soy milk compared to SPI-derived soy whey.

### 2.2 Soy oil processing byproducts

#### 2.2.1 Soy hull

Soy hulls are a byproduct of the soybean processing industry, primarily generated during the extraction of soybean oil (Fig. 1). These hulls consist of the fibrous outer covering of soybeans, typically accounting for about 5 % of the total weight of soybeans and are typically removed during the oil extraction process [7]. On a dry basis, soy hulls are mainly composed of dietary fiber (63.8–88.0 %) and protein (10–15 %) [37]. Due to their lightweight and voluminous nature, handling and transport require specialized infrastructure. While uncommon in human food products, soy hulls are valuable as ruminant feed, containing cellulose (29–52 %), hemicellulose (10–25 %), crude protein (11–15 %), pectin (4–8%), and lignin (1–4%) [42].

#### 2.2.2 Soy meal

Soybean meal is derived through various processing techniques, including solvent extraction and mechanical extraction from the soybean oil production process. Solvent extraction involves extracting oil from soybean flakes, resulting in residual oil content of approximately 1.5 %, while mechanical extraction using a screw press yields soy meal press cake with an oil content exceeding 5 % [7]. Soy meal constitutes 62.5 % of the total oil meal content and serves as a primary protein source, contributing 61 % to livestock feed [43]. Highly regarded for its nutritional value, soy meal is characterized by a crude protein, fat, fiber, ash, and carbohydrate content (Table 1). According to Messina [44] soy meal exhibits a more advantageous composition of essential amino acids, including methionine, arginine, leucine, isoleucine, lysine, tryptophan, valine, and others, compared to soybeans. In terms of fatty acid composition, soy meal exhibits relatively low levels of saturated fatty acids and contains minimal amounts of monounsaturated and polyunsaturated fatty acids [45]. Furthermore, soy meal contains sufficient quantities of calcium, iron, magnesium, phosphorus, potassium, sodium, zinc, and copper, as well as small amounts of water-soluble vitamins B1, B2, B3, pantothenic acid, B6, and folate [46].

#### 2.2.3 Lecithin

Lecithin, an endogenous compound, can be derived from various botanical and zoological sources such as egg yolk, leafy green vegetables, lean red meat, and sunflower seeds. It is widely used in the food and pharmaceutical sectors due to its exceptional emulsifying properties. A significant quantity of lecithin, a byproduct, is extracted through the degumming process during soy oil manufacturing (Fig. 1). Lecithin is separated during soybean oil refining to remove impurities that can affect the oil's stability, clarity, and shelf life. This also prevents issues like foaming, darkening, and off-flavors, making the refined oil more suitable for culinary and industrial uses. Soy lecithin, particularly, is valuable and cost-effective as it is extracted from soy oil industry byproducts [7]. Food manufacturers commonly utilize commercial soybean lecithin, which is a mixture of phospholipids. The composition of lecithin may vary depending on the source of soybeans. Predominant phospholipids found in soybean and sunflower lecithin include phosphatidylcholine (PC), phosphatidylinositol (PI), phosphatidylethanolamine (PE), and phosphatidic acid (PA). Importantly, lecithin is fully metabolizable by the human body, making it highly compatible for various dietary applications. Furthermore, lecithin is well-tolerated and poses no toxicity concerns upon ingestion [47,48].

## 3 Status and potential applications of okara

**Fig. 2:**
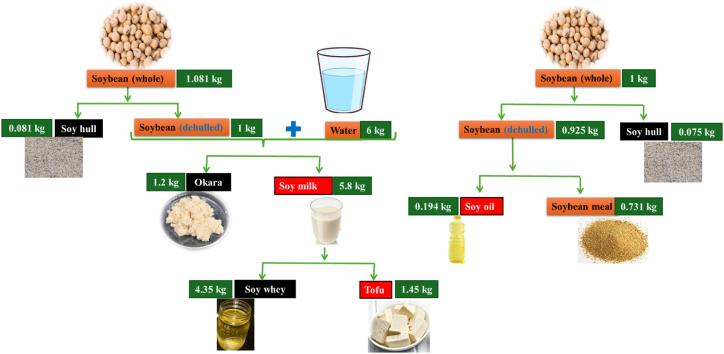
Waste generation during soy-based foods production and their byproducts.

The production of soy milk or tofu results in the formation of approximately 1.1–1.2 kg of okara for every kilogram of soybeans utilized (Fig. 2) [1,20,21]. For example, in the process of soy milk manufacturing, a combination of 1 kg of soybeans and 10 L of water is utilized, leading to the creation of 1.2 kg of okara [13,49]. Consequently, the okara generated during this process constitutes around 10.9 % of the utilized raw materials and is commonly disposed of without yielding any economic benefit. An analysis of processing costs for soy milk production shows that approximately $228 per metric ton of soybeans is lost in the form of okara byproduct [13]. In 2019, global soybean production totaled 342 million tonnes, with a portion allocated for tofu and soy milk production. This process generates approximately 14 million tonnes of okara annually, with major contributions from countries like China, Japan, and India [19,20]. Significant quantities of okara are produced annually in regions with high soybean consumption, especially in Asian countries. Hong Kong and Singapore are major consumers of soy milk both at regional and national levels, while countries like Australia and Canada also show substantial soy milk consumption [50]. The soybean curd manufacturing industry in Japan produces an estimated annual quantity of roughly 800 000 tons of okara. Similarly, in Korea, the annual production of okara by this sector is estimated at 310 000 tons. In China, the production of okara by the soybean curd manufacturing industry is significantly higher, estimated at 2 800 000 tons [51,52]. The annual production of okara in Singapore is estimated to be at least 10 000 tons, a quantity similar to the production level observed in Canada [53].

**Fig. 3:**
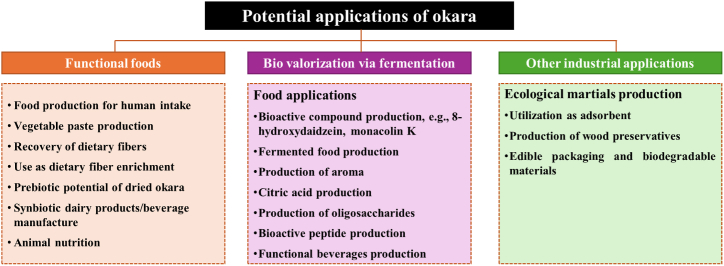
Potential valorization approaches to increase the utilization of okara.

Currently, okara is commonly considered a waste byproduct, with a substantial amount utilized for animal feed and organic fertilizer production. Colletti, Attrovio, Boffa, Mantegna and Cravotto [13] proposed that soy okara presents a cost-effective alternative to traditional soy meal in animal feed, with no discernible adverse effects on cattle health, offering potential economic and sustainability benefits. In addition, a lesser proportion contributes to traditional Japanese cuisine, featuring in dishes such as unohana, okara salad, and okara miso soup. Moreover, soy okara is a valuable raw material for producing a variety of plant-based protein products, including SPI, SPC, texturized soy protein, as well as the creation of meat substitutes and incorporation into baked goods [7]. Beyond its culinary applications, soy okara finds use in the production of probiotics, bio-okara, dietary supplements, and various industrial processes [13]. As shown in Fig. 3 numerous methods have been explored to maximize the utility of okara including its incorporation as a raw material for fiber production, lipid extraction, protein supplementation, ethanol manufacturing, and its potential in preventing diabetes, obesity, etc. [1,13,20]. Okara has also been integrated into composite flours for specialized dietary needs and functional foods, such as bread, pancakes, puffed snacks, noodles, candies, beverages, sausages, and nutritional flours [19,51]. To enhance the soluble fiber content of okara and improve its nutritional quality and functional properties, various techniques have been employed including chemical or enzymatic treatment, fermentation, extrusion, high pressure, and micronization [19,51,54]. Furthermore, novel mechanical treatments like high-pressure homogenization, high-speed mixing, and ultrasonics have successfully extracted fibers with enhanced functionalities from fibrous food byproducts such as okara [54].

Most tofu and soy milk facilities are small and widely distributed, making it difficult to collect enough okara for centralized processing before spoilage [55]. Okara's high moisture content (around 80 %) and rapid degradation make efficient drying technologies essential to stabilize it for further processing. However, designing cost-effective dryers that preserve okara's nutritional and functional qualities remains challenging [1]. Consequently, high drying costs and spoilage lead to substantial okara waste annually [1,3,13,56]. Moreover, the utilization of okara faces limitations due to its high perishability, low digestibility, undesirable sensory characteristics (fishy and beany flavor) reminiscent of soybeans, and an unappealing texture [19,57]. Additionally, it contains antinutritional factors, including raffinose oligosaccharides, enzyme inhibitors, lysinoalanine, phytic acid, tannins, and saponins [20,22,58]. While existing solutions show promise for achieving zero-waste objectives, challenges remain. Many rely on partial recovery of okara and involve energy-intensive drying, as well as additional inputs like enzymes, chemicals, or ingredients such as wheat flour [1,13]. Despite progress, optimizing okara preservation and creating value-added applications remains complex due to these limitations. To align with circular economy goals, it is essential to transform okara from a waste product into a resource. Developing innovative preservation and valorization techniques will be crucial for maximizing okara's potential, enabling sustainable, high-value uses that benefit both the environment and economy in the food sector [19,20].

## 4 Status and potential applications of soy whey

The current status of soy whey in the soybean food industry is characterized by its role as a predominant byproduct of processes involving soybeans, particularly the production of soy milk, SPI, and tofu [9]. Approximately 9 kg of tofu whey is produced for every kilogram of soybean used in the tofu process [59]. In the case of SPI, approximately 12 kg of soy whey is generated per 1 kg of SPI produced [60]. While research has extensively explored the utilization of okara as a dietary and nutritional component [1], the attention given to soy whey is comparatively limited, particularly on its potential in the realm of food and nutrition. Unfortunately, due to a lack of economically viable technology and insufficient incentives for recycling soy whey, a substantial portion is discarded untreated. This practice poses severe environmental risks, including unpleasant odors and pollution of surface and groundwater due to its high nutrient content and high biochemical oxygen demand (BOD). Typically, the BOD value for soy whey ranges from 10 000 to 20 000 mg/L [61]. In addition to environmental consequences, the discharge of soy whey into sewage systems results in the loss of valuable chemicals and nutritional components, notably soy isoflavones [7,29].

**Fig. 4:**
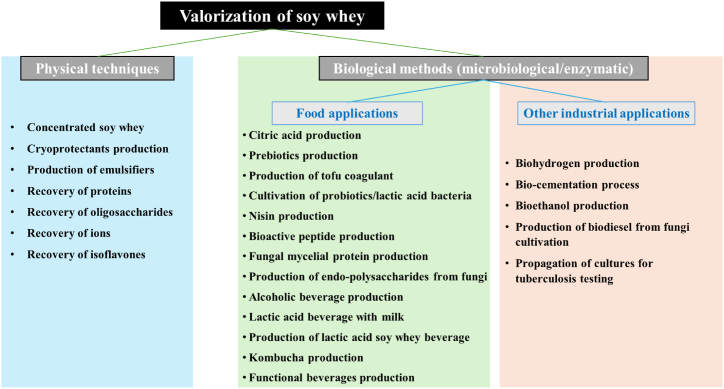
Potential applications of soy whey for different industrial purposes (Chua & Liu, 2019).

Various approaches to soy whey utilization have been explored, such as isolating its compounds, generating new ones through enzymatic or fermentation methods, developing functional beverages, and even synthesizing biofuels. Physical techniques primarily extract nutrients, while microbiological and enzymatic methods unlock its potential as a substrate for diverse food and non-food applications, broadening soy whey's usability across industries (Fig. 4). Extracting and processing isoflavones from soy whey has supported the development of popular isoflavone supplements due to their potential health benefits [9]. Converting soy whey to powder form could help reduce byproduct and create plant-based whey protein powders. However, high drying costs often limit this practice economically. Soy whey is also used to produce coagulants, prebiotics, citric acid, peptides, nisin, soy whey drinks, and alcoholic beverages, leveraging various feasible processing methods across food applications [9]. Furthermore, whey protein, amino acids, and oligosaccharides recovered from soy whey are subsequently employed in the production of emulsifiers, cryoprotectants, adhesives, and gelling agents [7]. The evolution of soy whey valorization has shifted significantly over time, moving from its primary use as a growth medium for microorganisms or bio-production to the direct development of novel consumer goods and industrial products. This transition highlights the versatility and increasing potential of soy whey across diverse applications [9].

## 5 Status and potential applications of soy hull

**Fig. 5:**
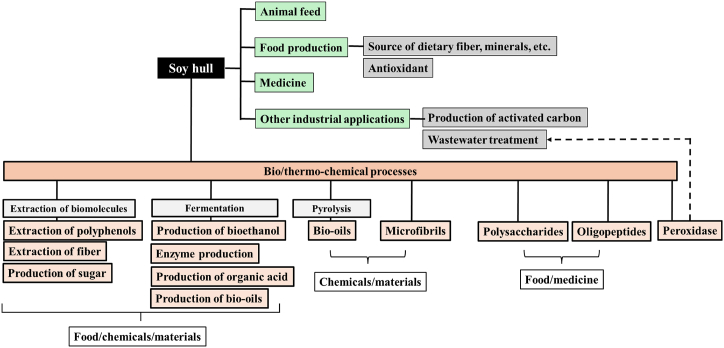
Possible applications and conversion of soybean hull.

Processing a ton of soybeans generates 50–80 kg of soybean hulls, which represents 5–8% of the weight of the whole soybean [37,62]. An estimated 18–29 million metric tons of soybean hulls were produced annually worldwide during the 2020/2021 period, based on annual global soybean production estimated at 359 million tonnes for this period [62,63]. Soy hulls are generally marketed in the form of granules and powder [63]. However, they are characterized by a rough taste and low nutritional value, making them primarily intended for animal feed, while considerable quantities continue to be thrown into landfills [37,63,64]. Singh and Krishnaswamy [7] reported that only a small portion, approximately 1 %, of the total soy hull products are dedicated to animal feed production. A substantial portion is allocated to produce nutritional fiber, ethanol, microfibril, bio-oil, and herbal medicine (Fig. 5). Other approaches to soybean hull valorization involve the exploration of sugars by lignocellulosic biorefineries for the production of valuable chemicals and biomolecules such as xylitol, butanol, itaconic, and succinic acid [64,65]. Recent research has also indicated that soy hull peptides may serve as an effective resource for wastewater treatment for adsorption of inorganic or organic pollutants [64].

## 6 Status and potential application of soy meal

**Fig. 6:**
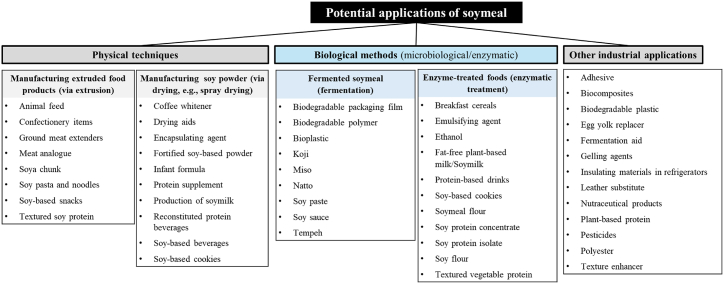
Potential applications and different valorization approaches of soymeal.

Singh and Krishnaswamy [7] illustrated that converting 10 lb (4.53 kg) of soybeans results in the production of 1.83 lb (0.83 kg) of soy oil and 8 lb (3.63 kg) of soy meal, representing approximately 80 % of the original weight of the soybeans. Soy meal holds a prominent position as a crucial source of nutrition in the animal feed industry, with approximately 37 million tons utilized for this purpose in animal agriculture. Within these allocations, broiler feed commands more than 45 % of total soy meal production, hogs account for around 19 %, and dairy animals contribute roughly 11 %. Turkey consumption represents over 8 % of soy meal usage, with the remainder allocated to feeding beef, sheep, goats, and aquatic animals. Studies show that the production of one ton of soymeal feed results in the release of 391 kg of CO_2_ equivalent greenhouse gases, contributing to global warming [66]. Soy meal plays a pivotal role in the United States economy through animal farming, making a substantial contribution of $165 billion in 2020 [7]. Notably, most of the soy meal (approximately 97 %) is allocated for animal feed purposes. The remaining 3 % is utilized for human consumption, primarily in the form of various soy-based products (Fig. 6), such as SPC, SPI, texturized soy flour, hydrolyzed energy concentrate, and crude protein concentrate [67,68].

Differences in the utilization of soy meal for human consumption can be influenced by various factors, including the presence of anti-nutritional constituents such as trypsin inhibitors, protease inhibitors, lectins, saponin, phytic acid, oligosaccharides, and antigens like glycinin and β-conglycin, as well as the absence of economically viable processing techniques. Reports suggest that fermentation induces chemical changes that reduce a significant portion of these anti-nutritional factors inherent in soy meal. Lactic acid bacteria, during fermentation, contribute to the reduction of trypsin inhibitors and phytase levels in soy meal. The duration of fermentation has been observed to impact the activity of anti-nutritional factors [25]. Despite soy meal being an economically efficient protein source, the expenses associated with essential processing to remove anti-nutritional elements substantially impact the overall cost of the final product. Moreover, soybean meal products exhibit distinct sensory attributes, characterized by a blend of beany, malty, and painty flavors, which significantly influence consumer preferences and acceptability. These sensory characteristics play a pivotal role in product development and marketing within the soybean meal industry [69]. Overall, soy meal presents substantial potential as an economically viable plant-based protein source capable of fulfilling the nutritional requirements of various age groups.

## 7 Status and potential application of lecithin

**Fig. 7:**
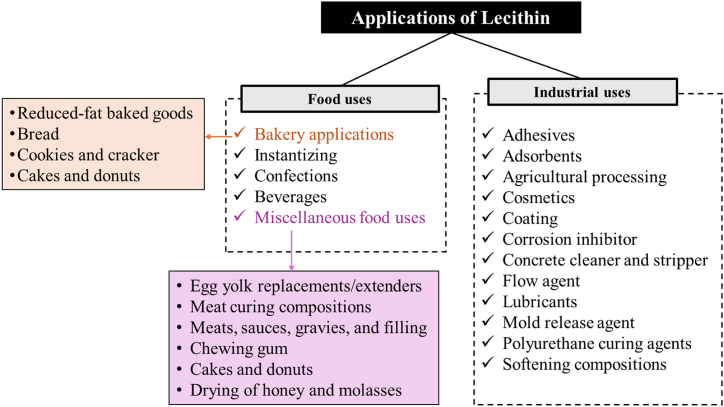
Potential applications of soybean lecithin in food and other uses.

Lecithin plays a crucial role in enhancing the stability of fats and improving the texture of various food products (Fig. 7), including salad dressings, desserts, margarine, chocolate, baked goods, and cooking preparations [70]. Lecithin offers medicinal attributes that contribute to improving cardiovascular health by reducing cholesterol and low-density lipoprotein levels in the body [71]. Additionally, lecithin provides various other health benefits, such as enhancing digestive capabilities, cognitive performance, immune system functionality, and potential assistance in preventing gall bladder and liver ailments. According to research by Alhajj, Montero, Yarce and Salamanca [72], several studies suggest that lecithin may serve as a beneficial preventive measure against physiological diseases such as bipolar disorder, anxiety, eczema, and various skin ailments. This multifaceted nature of lecithin underscores its significance in both the food industry and healthcare, making it a valuable and versatile compound with numerous potential applications. In addition to its use in human food products, soybean lecithin finds diverse applications across various industries. It serves as a release agent for plastics, an anti-gumming agent in gasoline, and functions as an emulsifier, spreading agent, and antioxidant in several industrial sectors, including textiles and rubber. Soybean lecithin, known for its composition of polar molecules, typically contains two reactive elements (P and N) incorporated into its molecular structure. This unique composition makes lecithin a highly favorable choice for lubricants, effectively reducing wear and pressure [48].

In relation to vegetable-derived lecithins, the process of extracting them is predominantly linked to vegetable seeds. Nevertheless, prior studies have provided evidence of the possibility of extracting substances from fruit pulp, as exemplified by research conducted on avocado and olives [73,74]. A variety of plant sources are available for the extraction of lecithin, with soybeans being the most extensively studied and documented. Other notable sources include sunflower, canola, corn, rice bran, and cottonseed. It is of significance to mention that, as reported by the U.S. Food and Drug Administration, genetically modified soybeans constituted 94 % of the total soybean cultivation in the United States in 2018 [72]. This statistic holds special importance when considering the utilization of lecithins derived from non-genetically modified origins [75]. As previously stated, phosphatidylcholine is the primary phospholipid found in all plant sources, with phosphatidylethanolamine and phosphatidylinositol following suit. The relative amounts of these phospholipids vary depending on the specific source. Like other soy byproducts, lecithin valorization faces several challenges, including high extraction and purification costs, quality consistency issues, and fluctuating market demand. The technical complexity of processing and the environmental impact of waste management further complicate its economic viability. Additionally, maintaining stability and proper storage for lecithin, especially in liquid form, adds to the processing difficulties [76,77].

## 8 Towards sustainable valorization: A framework for zero-waste soybean processing

### 8.1 Limitations for sustainable solutions in soybean byproduct valorization

Soybeans are esteemed as a superfood due to their exceptional nutritional value. Primarily utilized for soy oil production (85 %) and animal feed (13 %), with only a small fraction (2 %) designated for human consumption [78]. Notably, the processing of soybeans yields substantial byproducts, such as okara and soy whey, alongside soy meal. Despite their high protein and nutritional content, a significant portion of these byproducts is underutilized or wasted [9,12]. To address this, there is a burgeoning interest in developing nutritionally rich foods for human consumption from soybean byproducts. However, challenges such as microbial contamination and short shelf life due to high moisture content need to be overcome, especially with okara and soy whey [13]. Additionally, soy meal presents flavor and anti-nutritional factor concerns. Therefore, sustainable approaches are imperative to harness the potential of these byproducts. Drying technologies, particularly spray drying, offer a viable solution to reduce moisture content, mitigate anti-nutritional factors, and enhance flavor while preserving bioactive components [79,80]. This processed soy powder can serve various purposes, including plant protein supplements, infant formula, and encapsulating material for bioactive components. In general, vegetable proteins, including soy protein, denature more easily than milk proteins during spray drying. This denaturation affects the solubility and functional properties of the soy powder. Formulating protein powders with wall materials like maltodextrin could enhance their reconstitution and handling properties. Additionally, hydrolyzing the protein prior to spray drying can boost solubility and foaming ability [81].

Despite efforts to valorize soy byproducts, the current technologies and methods used often result in the generation of substantial waste. Although a portion of these byproducts is recovered and utilized, a significant amount of waste is still produced in the process. This necessitates a continual pursuit of novel strategies to fully exploit the potential of soybean processing byproducts and address emerging challenges in the industry. By maximizing the utilization of soybean processing byproducts, industries can achieve total valorization of waste through innovative approaches such as advanced fermentation technologies, enzymatic treatments, and comprehensive recycling methods. This holistic byproduct valorization approach contributes to the establishment of a zero-waste, sustainable bioprocess system, ultimately resulting in a more environmentally friendly and economically viable production chain.

### 8.2 Innovative approaches for sustainable soybean processing

**Fig. 8:**
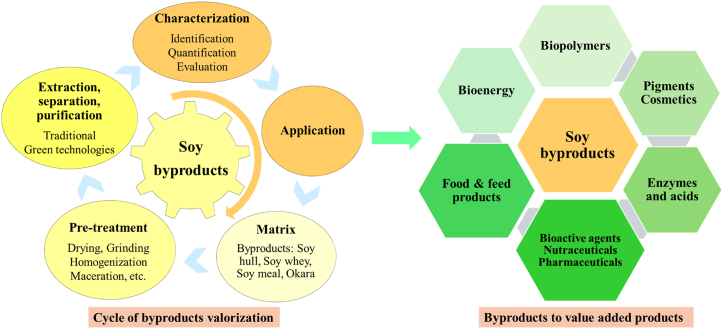
Comprehensive valorization cycle of soy byproducts into value-added products for diverse industrial application.

The soybean processing industry can move towards a zero-waste paradigm by adopting innovative strategies that ensure all byproducts are valorized, optimizing resource use, and supporting a sustainable economy. Achieving this goal requires integrating sustainable valorization practices, circular economy principles, and advanced technologies such as biorefineries. Fig. 8 illustrates a comprehensive valorization cycle for soy byproducts, including pre-treatment, extraction, and application steps that transform materials like soy hulls, whey, meal, and okara into valuable products. These value-added products span diverse sectors, such as bioenergy, food and feed, biopolymers, bioactive agents, and pharmaceuticals. The framework presented emphasizes strategies for fully utilizing soy byproducts, particularly in food and feed applications, thereby enhancing sustainability within the soybean industry.

#### 8.2.1 Maximizing okara utilization in diverse industries

**Fig. 9:**
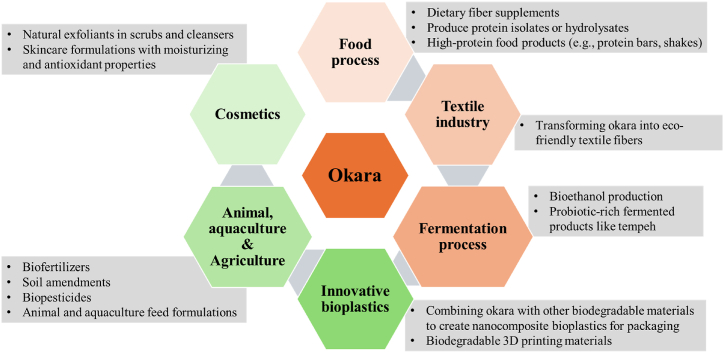
Expanding okara utilization across diverse industries to transform it into high-value products to minimize waste production from soy beverage manufacturing.

Okara contains essential nutrients and is gluten-free, presenting an opportunity for further valorization [1,82]. For examples, developing processes for soy beverage production could effectively utilize okara. Plant-based beverages have a long history of consumption, serving as both traditional drinks and dairy milk alternatives [83]. Plant-based alternatives, such as soy, oat, almond, rice, and coconut beverages offer benefits like being cholesterol-free and lactose free, catering to diverse dietary needs [84,85]. Plant-based beverages are witnessing a surge in popularity, particularly among vegetarians, driven by ethical, environmental, and health concerns [83,86,87]. Drawing on lessons from the broader plant-based beverage industry, the soy-based beverage sector can be expanded into a promising industry for diverse new soy-based beverages and food applications such as soy-based protein shakes, soy lattes, soy smoothies, flavored soy milks, fermented soy drinks, and soy-based energy drinks. Additionally, maximizing okara utilization can be expanded to diverse industries by converting it into high-value products (Fig. 9). By effectively utilizing all components of okara across diverse industries, waste production from soy beverage manufacturing can be minimized by transforming a byproduct into high-value products.

Okara, a byproduct of soy beverage production, has significant potential as a functional food ingredient, as it can replace wheat and soy flours in food products, enhancing their fiber and protein content. Guimarães, Silva, Lemes, Boldrin, da Silva, Silva and Egea [49] conducted an economic analysis estimating that the byproduct from soy beverage manufacturing makes up 10.9 % of raw material usage, leading to a raw material loss valued at approximately $227.7 per ton of okara. By incorporating okara into foods, manufacturers can create value-added products, thereby reducing waste and environmental impact. Thus, a significant economic value can be added to the waste by generating a new food product or food ingredient. Studies have also examined processing techniques to optimize okara's nutritional benefits. Lee, Gan and Kim [88] used thermal treatment and fermentation with Rhizopus oligosporus before drying to produce an okara flour suitable for enhancing functional characteristics in foods like biscuits, with consumers accepting okara substitutions up to 20 %. Similarly, Lu, Liu and Li [89] used okara to replace 10–25 % of wheat flour in bread and noodles, producing items with a lower glycemic index, which could aid in diabetes prevention. However, these substitutions affected sensory qualities such as loaf volume and flavor, indicating the need for further refinement to balance nutrition with desirable taste and texture in food applications.

#### 8.2.2 Maximizing valorization potential through value-added product extraction

Novel extraction methods could enable the recovery of biopolymers, including proteins, polysaccharides, and lipids, from soybean processing residues. These biopolymers offer versatile applications in industries such as food packaging, biomedical materials, and biodegradable plastics, contributing to sustainable innovation across various industries [19,62]. Additionally, there is an opportunity to develop innovative processes and technologies for extracting specific value-added products, such as phytosterols, from soybean oil deodorizer distillate (DOD), a byproduct of oil refining [90]. Phytosterols offer diverse health benefits and can be seamlessly integrated into functional foods, dietary supplements, or pharmaceuticals. Moreover, it is crucial to explore cutting-edge technologies for converting soybean hulls into value-added products like dietary fiber, biofuels, or bio-based chemicals. Advanced processing techniques such as mechanical pretreatments, enzymatic hydrolysis, fermentation, pyrolysis, or their combination can be employed to fully exploit the valorization potential of soybean hulls. Okara can be also upcycled into nutritional extracts for micronutrient encapsulation, such as β-carotene, to address micronutrient malnutrition in developing countries [91]. Additionally, extracting prebiotic fibers, protein concentrates, and peptides from okara for functional food applications, along with bioactive compounds such as isoflavones, saponins, and antioxidants for nutraceutical applications, significantly enhances the valorization potential of soy byproducts. In recent years, the market for functional foods, particularly prebiotics, has grown significantly. In 2015, the global prebiotics market was valued at over $2.9 billion, with projections estimating a growth rate of approximately 12.7 % by 2025, reaching an estimated value of $10.55 billion [92]. The dietary fiber modifications in okara were also aimed to improve texture and flavor by increasing soluble dietary fiber (SDF) content, enhancing compatibility with food matrices. Methods such as micronization [93], wet grinding with thermal pretreatment [94], sonication [95], and cavitation jet processing [96] have been shown to enhance water holding, oil holding, and swelling capacities. Additionally, Wei, Ye, Li, Wang, Li and Zhao [97] developed a layer-by-layer chitosan/pectin coating for okara insoluble dietary fiber, improving its textural properties and sensory quality in tofu.

#### 8.2.3 Complete valorization for a zero-waste system

Complete valorization of soybeans, without generating byproducts like okara, represents a sustainable approach to soybean processing. The current standard practice often involves partial valorization, where soybean byproducts like okara are repurposed as animal feed, incorporated into baked goods, or used in vegetarian and vegan food products [9,78]. While these partial valorization methods reduce waste and can provide additional revenue and utility, they do not fully unlock the complete potential of these byproducts, and residual waste is often generated. In contrast, the concept of complete valorization may represent a more comprehensive and forward-thinking approach that can strive to eliminate waste, ultimately benefiting both the industry and the environment by maximizing the value extracted from soybean byproducts. Future research efforts for this approach requires the development of innovative processes and technologies that can transform these byproducts into high-value products or seamlessly reintegrate them into the primary production process. In soybean processing, complete valorization aims to convert okara into additional soy-based products or integrate it into the soy milk production process, reducing waste and maximizing resource use. This approach enhances efficiency and sustainability by using okara at various production stages, creating a zero-waste system that fully utilizes all soybean components. Examples include repurposing okara to enhance soy milk quality, enriching nutritional content, or developing new soy-based food products, thus achieving circularity in the soybean industry. Several practical examples of how to valorize complete soybeans without producing waste.

**Fig. 10:**
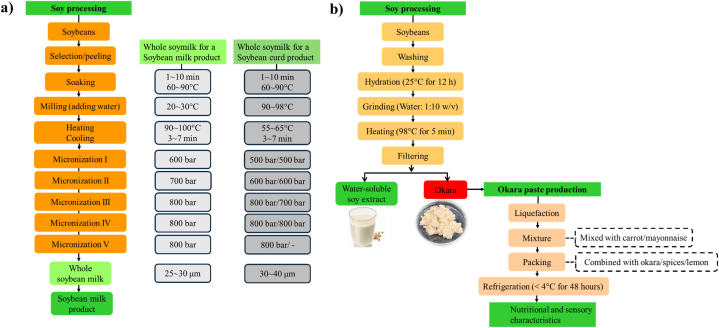
Flow diagrams for (**a**) a method of producing ‘whole soybean milk’ and curd that retains the full nutritional profile of soybeans while eliminating waste byproducts [99], (**b**) a method of utilizing okara for producing a vegetable paste, a homogeneous blend of vegetables enhanced with additional flavorings to improve sensory attributes [49].
• Traditional soy milk production for tofu involves milling and heating soybeans, resulting in a product with about 10–13 ⁰Brix concentration. However, creating highly concentrated soy milk (approximately 21 ⁰Brix) is challenging due to the need for expensive equipment, time consumption, and increased bacterial growth. Hoshi, Takada and Chitose [100] produced a highly concentrated soybean milk paste that retains all soybean nutrients, minimizes bacterial contamination, and is cost-effective. This is achieved by mixing soybean powder, milled soybeans, or soybean flakes with water to create a fresh mixture at 21 ± 4 ⁰Brix concentration, followed by boiling, heating, and cooling. This process can be done using a heat-resistant bag or retort pouch to ensure pasteurization and reduce the growth of bacteria without requiring expensive machinery.
• Lim and Choi [101] proposed an efficient method for producing whole soybean milk with fine particle size and enhanced storage stability, achieving this without generating any waste byproducts. Unlike conventional methods that result in significant nutrient loss and produce undesirable textures, this method involves roasting and dehulling soybeans, cooking them, coarsely grinding the cooked soybeans, finely grinding the particles in a cutting manner, and homogenizing the resulting whole soybean liquid. This process yields whole soybean milk with stable viscosity, extending its shelf life and retaining the entire nutritional content of the soybeans.

• A conventional process for preparing soybean milk or curd involves heating and milling soybeans and pressing the resulting slurry, which discards about one-third of the soybean feed as solid residue, wasting nutrients. Various high-pressure processes have been developed to micronize particles in soybean milk, including high-pressure homogenization and high-temperature treatment followed by high-pressure micronization [85,98]. Cho and Oh [99] introduced a method for producing ‘whole soybean milk’ and curd that retains the full nutritional profile of soybeans while eliminating waste byproducts (Fig. 10a). This method involves milling soybeans and homogenizing them through at least two steps of ultra-high pressure micronization, with each step applying at least 500 bar and a total cumulative pressure of at least 2000 bar, eliminating the need for extra steps like enzymatic treatments [99].

#### 8.2.4 Simultaneous valorization approach

Two strategies are possible for utilizing soybean residues: (1) incorporating soybean residues into existing products to enhance their properties, and (2) combining two or more types of soybean residues to create new functional foods. The utilization of soybean pulp residue, or okara, has been explored for its potential as a highly beneficial component in various food applications, including pastes, processing aids, and food fortification [1,102]. One example of its utilization is in the production of vegetable paste (Fig. 10b), a homogeneous blend of vegetables enhanced with additional flavorings to improve sensory attributes [49]. The process involves liquefying carrots and mayonnaise to create a uniform mixture, which is then combined with soybean pulp, spices, and lemon juice in appropriate proportions to achieve a product that is both well-received and nutritionally balanced. The authors conducted an economic analysis of okara, a byproduct of water-soluble soybean extract production, revealing that 10.9 % of the raw material, valued at approximately $227.7 per tonne, is typically discarded. Incorporating okara into food products not only reduces disposal costs and environmental impact but also adds economic value by repurposing waste into a valuable ingredient for food enrichment. Similarly, okara can be valorized concurrently with various food byproducts, including fruit pulp residue (such as apple pulp, orange pulp, and grape pulp), vegetable pulp residue (including carrot pulp, tomato pulp, and potato pulp), grain pulp residue (like wheat pulp, barley pulp, and corn pulp). This approach aims to develop innovative food products with enhanced nutritional value and sensory characteristics [103,104].

Currently, there is a noticeable increase in demand for plant-based beverages and their byproducts, primarily driven by vegetarian consumers [83]. In this sense, okara and soy whey can be concurrently valorized to create innovative drinks and beverages through appropriate technologies. Apart from simultaneous valorization of byproducts, a novel combination of different technologies can also be employed to obtain improved nutritional value and functional foods. For example, Pérez-López, Mateos-Aparicio and Rupérez [105] observed a synergy between high hydrostatic pressure (HHP) and Viscozyme® L treatment, enhancing SDF release and decreasing molecular weight to maximize SDF content. HHP, a novel process, increases the value of byproducts like okara by solubilizing their dietary fiber. Some enzymes, including Viscozyme® L, show increased activity under HHP. Herein, an optimized simultaneous method with Viscozyme® L and HHP reduces industrial processing costs. A study on the modification of SPI through combined extrusion pre-treatment and controlled enzymatic hydrolysis demonstrated that extrusion significantly enhanced SPI's accessibility to enzymatic hydrolysis, yielding improvements in degree of hydrolysis, protein solubility, and surface hydrophobicity, thereby enhancing the emulsifying properties of the treated soy protein hydrolysates [106]. Similarly, ultrasound-assisted glycosylation enhanced the functional properties of SPI [107,108].

#### 8.2.5 Closed-loop byproduct management and collaborative supply chain

In sustainable soybean processing, thorough identification and characterization of all generated byproducts like okara, soy whey, and soy hulls are crucial. Analyzing these byproducts at their source helps understand their composition and nutritional value, facilitating efficient management and potential utilization [7,13]. Integrating valorization processes back into the soybean processing chain could establish a closed-loop system, minimizing waste and maximizing resource efficiency. Continuous monitoring and optimization of this system are vital for enhancing efficiency, reducing environmental impact, and bolstering economic viability.

One approach to create a closed-loop byproduct management system without producing waste can be like the method of Kim and Kim [109], who proposed a system for producing whole soy milk using both soybeans and their skins. This method involves washing and drying the beans, transforming their starch into water-soluble dextrin using a heat chamber, and grinding them into ultra-fine, nano-scale particles. The resulting mixture is then homogenized and sterilized, ensuring improved digestion, taste, and elimination of malodors. This process retains all the nutritional components of the beans, including valuable fibers and anti-aging compounds, while avoiding waste. Additionally, the invention offers a cost-effective, high-quality alternative to cow milk for various dairy products. Another approach is to utilize all generated byproducts, such as okara, soy whey, and soy hulls, within the industry for value-added products. For example, okara is produced from soy milk production, while soy whey is generated during tofu production. Both okara and soy whey can be used simultaneously or separately as raw materials for other valuable product production following a biorefinery concept [110,111]. Thus, no waste will be produced from any industry, and all byproducts will be valorized within the industry. However, it is essential for researchers to perform a cost-benefit analysis for each technology presented to assess its economic feasibility at the commercial level to determine if the additional step required for technology implementation is viable in a commercial setting.

**Fig. 11:**
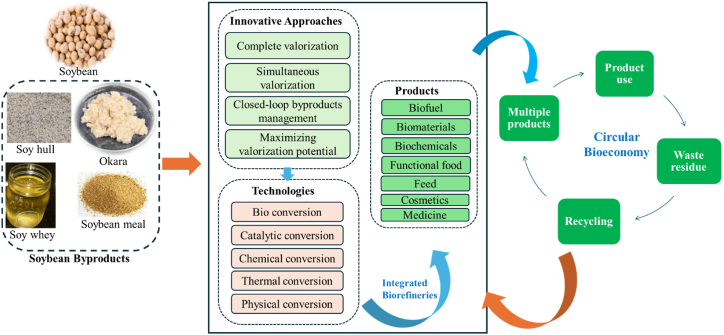
A circular bioeconomy framework for zero-waste soybean processing, showcasing the complete utilization and valorization of byproducts.

A conceptual framework for zero-waste soybean processing is summarized in Fig. 11, which illustrates the circular bioeconomy approach for soybean processing, highlighting the complete utilization of byproducts such as okara, soy whey, and soy hulls. The framework emphasizes the transformation of waste residues into valuable products through stages of product use, recycling, and the generation of multiple products, ensuring no waste is produced and all byproducts are efficiently valorized within the industry.

## 9 Prospect and challenges of processing soybean byproducts

### 9.1 Anti-nutrients and allergens

Soybeans, in their unprocessed state, contain notable levels of anti-nutritional factors, particularly trypsin inhibitors, which undergo partial deactivation during solvent extraction and roasting procedures [112]. Trypsin inhibitors are a significant factor limiting the use of okara in animal feed due to their adverse effects on cattle digestion, which also hinders its application in animal research. Many trypsin inhibitors also inhibit chymotrypsin, affecting overall protein digestion by blocking these critical digestive enzymes. Experimental findings indicate that the utilization of raw okara as direct feed affects growth, behavior, and physiological activity in animals [113]. Soybeans also contain anti-nutritional factors like lectins, which can interfere with protein and carbohydrate digestion.

These effects can be minimized or eliminated through appropriate processing methods [114,115], including physical (e.g., grinding, high-pressure homogenization (HPH), milling, soaking, ultrasonication), chemical, bioreduction, thermal (e.g., autoclaving, blanching, boiling, cooking, drying, extrusion, microwaving, roasting, steaming), and biotechnological (e.g., enzymatic, germination, fermentation) methods [112,116,117]. For example, fermented okara shows a reduced presence of anti-nutritional components and minimal quantities of vitamin B-2, vitamin B-12, and flavoproteins [9]. For soybeans, heating at 100 °C for 9 min effectively inactivates most trypsin inhibitors [118]. Similarly, autoclaving soybean flour for 5 min reduces over 90 % of lectins and trypsin inhibitors, while preserving protein solubility above 80 % [119]. However, excessive heat processing can lead to protein damage, decreasing protein quality and negatively affecting properties like solubility. It may also degrade essential heat-sensitive vitamins. Therefore, selecting the appropriate heating duration and intensity is essential. Li, Zhu, Zhou and Peng [120] demonstrated that treating soy proteins with high hydrostatic pressure (HHP) at 300 MPa for 15 min reduced allergenicity by approximately 48.6 % compared to untreated SPs. This reduction is likely due to conformational changes and the breakdown of antigenic sites during HHP processing, suggesting potential for HHP-treated SPs as hypoallergenic ingredients in infant formulas [121]. Genetic and breeding strategies to develop cultivars with low anti-nutritional factors could be effective and practical. For example, plant breeding has successfully reduced saponin content in soybeans [122]. Recent advancements, such as CRISPR/Cas9 gene editing, are now being used to suppress the biosynthesis of these factors, leading to the creation of saponin-free plants [123]. Additionally, it is worth noting that soy meal consists of complex carbohydrates, specifically non-starch polysaccharides, which serve as essential dietary fibers but can interfere with the digestion and absorption of various nutrients present in food (Yasothai, 2016). Therefore, meeting regulatory standards and ensuring food safety in the production of soybean byproduct-derived foods is challenging and requires compliance with various food safety regulations and quality standards.

### 9.2 Soybean odor and raw beany smell

Soy milk, rich in high-quality protein, linoleic acid, vitamin E, and isoflavones, faces acceptance challenges due to off-odors such as raw and beany smells, mainly caused by lipoxygenase activity. Methods like cooking, high-temperature grinding, and using lipoxygenase-deficient soybeans have been proposed, but none entirely resolve the issue. Off-odor compounds, including hexanal, hexanol, and (E,E)-2,4-decadienal, arise from the oxidation of unsaturated fatty acids [[124], [125], [126]]. For example, roasting soybeans at 200 °C for 20 min effectively inactivated lipoxygenase and peroxidase, but it also led to a significant reduction in the content of essential amino acids and protein solubility [127]. Though heat treatment remains the most effective method to remove these odors, several multi-method approaches offer promising solutions for enhancing sensory quality [128]. For example, mixed fermentation with lactic acid bacteria (LAB) and kombucha bacteria enhances the nutritional profile of soy milk by increasing levels of isoflavones and B vitamins, such as riboflavin and cobalamin. This process also reduces the characteristic "beany" smell and introduces new flavors, enriching the overall taste profile [129]. Poliseli-Scopel, Hernández-Herrero, Guamis and Ferragut [130] demonstrated that soy milk treated with ultra-HPH at 200 MPa had lower microbial populations, reduced beany flavor and astringency, and improved colloidal stability compared to pasteurized (90 °C for 30 s) soy milk during storage. Other non-thermal treatments, such as ultrafiltration, have also been applied in soy product production [131]. In a related study, a chocolate-flavored soy beverage made with cocoa powder scored significantly higher in sensory ratings for taste, odor, texture, and overall acceptance, establishing it as the preferred choice among panelists. These findings highlight cocoa powder's effectiveness in masking off-flavors, thereby enhancing the sensory profile of soy-based beverages [132]. Cyclodextrins, particularly β-cyclodextrin, effectively mask the beany flavor of soybeans by forming inclusion complexes with odor-causing compounds. Studies have shown that α-, β-, and γ-cyclodextrins can significantly reduce soybean off-flavors, with α-cyclodextrin removing over 95 % of odor precursors, while β-cyclodextrin reduces key beany flavor compounds such as 1-octen-3-ol, benzaldehyde, hexanal, and 2-heptaneone [133,134]. Takagi and Hikichi [125] proposed a method to produce an improved soy milk with a pleasant flavor and reduced soybean odor, raw smell, and astringency. The method involves mixing soy milk with a triacylglycerol containing medium-chain fatty acids (6–12 carbon atoms) to achieve a content of 5–15 mass % relative to the soy milk mass. This addition effectively improves the flavor by suppressing the undesirable tastes and odors associated with soy milk. Furthermore, rosemary (*Rosmarinus officinalis*) extract effectively inhibits lipid oxidation and reduces rancid and beany off-flavors in soy milk enriched with fish oil, enhancing both the oxidative stability of lipids and the organoleptic quality of the soy milk [135]. As mentioned, soy-based products are often supplemented with artificial additives to improve their nutritional and sensory qualities but blending them with natural plant or animal-based ingredients is becoming a popular trend [136]. Examples include mixing soy beverages with fruit juice [137], cereals and legumes [138], and okara [139], as well as preparing soy yoghurt-cheese from a combination of soy milk and cow milk [140].

### 9.3 Concentration and drying

While nutrient extraction from soy whey shows promise, its commercial viability is limited due to low nutrient concentrations—typically around 1 % per nutrient class. Effective valorization would require concentration techniques to amplify nutrient levels by 8–32 times, which incurs significant costs [9]. Therefore, conducting detailed cost-benefit analyses is essential to determine whether these additional processes can be economically integrated into large-scale soy whey valorization. Another aspect is that in the dairy industry, before spray drying, milk is concentrated by evaporation from 12 % to 50 % total solids, making the drying process economically viable [141]. For soy milk, the initial total solids content is around 7–10 % [142]. Concentrating soy milk through evaporation or filtration is challenging due to high viscosity, which limits total solids to about 22 % before membranes block or milk burns on evaporator walls. Concentration for soy milk must be kept lower than for cow's milk to avoid excessive viscosity issues. While traditional milk powder spray-drying equipment can be adapted for soy milk, modifications are needed. Attempts have been made with ultrafiltration membranes to concentrate soy milk. Additionally, fluidized bed drying using inert particles has been developed as a lower-cost alternative to spray-drying, delivering comparable product quality [143,144]. To make the drying process for soy milk economically feasible, innovative technologies are still needed. Future research could explore methods to reduce soy milk viscosity for improved processing efficiency. Strategies may include: (i) employing enzymes like cellulases and pectinases to break down complex carbohydrates, thereby reducing viscosity; (ii) adding hydrocolloids such as carrageenan or guar gum in precise amounts to modify texture without compromising quality; (iii) optimizing pH levels to naturally reduce viscosity; (iv) applying controlled heat treatments to alter protein structure, lowering viscosity; and (v) using high-shear mixing or homogenization to disperse particles, effectively reducing viscosity. These approaches could collectively enhance soy milk processing while maintaining product quality [143,[145], [146], [147]].

According to Vong and Liu [1], advancements in the soy beverage industry—such as high-heat treatments and the use of sanitary processing equipment under the HACCP system—enable the production of okara with lower microbial contamination, making it a safer substrate for bio-valorization. Studies have explored various drying techniques and treatments to optimize okara flour, highlighting how different processing conditions can significantly impact its properties, nutrient composition, and phytochemical content [82,148]. Dried okara has shown potential as an ingredient substitute in products like bread, biscuits, cakes, and noodles, offering a nutritious, sustainable alternative in food production [88,149]. However, drying okara is usually energy-intensive due to its high-water content [1,13]. Economic analysis has shown that the cost of drying okara greatly exceeds the value of the protein it contains [51].

### 9.4 Viscosity and solubility of soy powder

The viscosity and solubility of powdered soy products pose significant challenges in various food applications [150,151]. Inadequate processing of soy powder can result in inconsistent viscosity levels when mixed with liquids, thereby affecting its suitability for specific uses such as beverages or food formulations. For example, protein solubility is essential for producing soy-based infant formula that is uniform, easily mixable, and nutritionally complete. Proteins with low solubility may not mix well with other ingredients like lipids and carbohydrates, leading to separation, precipitation, and inconsistent quality. This inconsistency can increase energy usage, production time, and costs due to issues during spray drying, as well as clumping and sticking in packaging, which reduces infant formula storage stability [152]. For consumers, protein solubility directly affects the reconstitution of infant formula, with poorly soluble proteins forming clumps or sediment, potentially causing digestive discomfort in infants. Additionally, incomplete solubilization can increase allergy risks, as undissolved protein fragments may trigger immune responses [153]. The solubility of soy powder could be improved using different innovative processing techniques like enzyme catalysis, glycosylation, HHP, high-intensity ultrasound, micronization, flash drying, and the use of capping agents, etc. [117,154]. Li, Zhu, Zhou and Peng [120] demonstrated that treating soy proteins with HHP at 200–300 MPa for 5–15 min enhanced their solubility, water-holding capacity, emulsification activity, and foaming ability. This improvement is attributed to the unfolding of the SPI structure, which enhances protein-solvent interactions and consequently increases solubility. In another study, high-intensity ultrasound treatment (20 kHz, 400 W, for 5–40 min) significantly reduced particle size and turbidity of soybean β-conglycinin, enhancing solubility, emulsifying activity, and emulsion stability [155]. This improvement in solubility is attributed to the disruption of protein aggregates, the unfolding of the protein structure, and increased surface hydrophobicity, which enhances protein-solvent interactions [156].

### 9.5 Dairy vs plant-based products

Producing plant-based dairy substitutes that replicate the nutritional and sensory qualities of dairy is challenging due to significant differences in chemical composition. Additionally, plant sources like soy and legumes often impart a distinct 'beany' flavor, which is less appealing compared to the taste of conventional dairy [70]. Antinutrients like inositol phosphate found in these plant-based materials can hinder mineral absorption and degrade nutritional value [157,158]. To address the shortcomings of plant-based dairy analogues, various additives such as stabilizers, fillers, nutrients, and processing aids are commonly employed [158,159]. For instance, oils are added for flavor and texture, while lecithin aids in emulsion stabilization [70]. These analogues are often fortified with vitamins (e.g., A and D) and minerals (e.g., calcium) to align with the nutritional content of bovine milk [70]. However, this can diminish their appeal to consumers who seek clean-label products and are concerned about the flavor and nutritional issues associated with highly processed plant-based foods [160,161]. Consequently, current research is increasingly focusing on fermentation as a potential solution to address the limitations of dairy substitutes while reducing reliance on additives. Numerous studies have highlighted the benefits of fermentation for plant-based dairy alternatives, including antinutrient reduction, sensory enhancement, and off-flavor mitigation [70,159]. Furthermore, fermentation enhances the bioactivity of polyphenols by transforming them into more potent compounds. Polyphenols can promote the growth of beneficial microorganisms and inhibit harmful ones, improving gut health and the nutritional profile of foods [162,163]. For instance, fermentation of whole soybean flour enhances the concentration of bioactive aglycones, such as daidzein and genistein, which possess notable antioxidant properties and health-promoting effects, including anti-inflammatory and anti-cancer activities [164]. Driven by accelerated aging and growing awareness of the link between diet and health, the global polyphenol market is projected to reach $1.82 billion by 2025, reflecting a growth rate of 7.44 % from 2020 to 2025 [165]. Additionally, fermentation enhances the sensory and functional properties of soy products by breaking down larger macromolecules (carbohydrates, proteins, and lipids) into smaller compounds like sugars, peptides, amino acids, and fatty acids [166]. The process also affects isoflavone levels, with longer fermentation periods (e.g., meju and doenjang fermented for 6 months with *B. subtilis* and *Aspergillus*) resulting in higher concentrations of peptides, amino acids, and other breakdown products compared to shorter fermentations (e.g., chungkukjang fermented for 72 h with *B. subtilis*) [166,167]. Herein, ensuring that products derived from soybean byproducts meet consumer preferences and align with nutritional expectations is essential for market success.

### 9.6 Learning from dairy whey: A parallel approach

Learning from dairy whey utilization offers insights into efficiently utilizing soy whey to minimize waste. Similarities in applying dairy whey to produce alcoholic beverages and LAB-fermented dairy whey beverages can inform current research on soy whey fermentation as a waste reduction technique [168,169]. A soy-based alcoholic beverage was produced by fermenting tofu whey with *Saccharomyces cerevisiae* strains, yielding an ethanol content of 7–8%. During fermentation, isoflavone glucosides were hydrolyzed into bioactive aglycones, enhancing antioxidant capacity. The process also generated volatile compounds, particularly esters and higher alcohols, which contributed fruity and floral aromas to the beverage [169]. However, optimizing soy whey fermentation for such applications is complex, involving challenges like managing fermentation parameters and considering unique composition of substrate. Recent research conducted by Tu, Tang, Azi, Hu and Dong [30] has expanded the scope of soy whey fermentation beyond alcoholic beverages to explore kombucha production, offering adaptability and potential of this method. However, challenges related to scaling up and ensuring product consistency in commercial production settings need to be addressed. A pilot study scaled foam fractionation of soy whey proteins to a 120 L system using soy whey, revealing that the process—especially foam fractionation and thermal sterilization—decreased food-related properties by approximately 40 %. Despite a recovery rate of 30.6 %, up-scaling slightly reduced whey proteins recovery and increased protein denaturation, impacting properties like solubility, foaming, and emulsification [31]. Moreover, several studies have aimed to convert 100 % soy whey into valuable products, including functional soy whey beverages utilizing strains like *Lactobacillus plantarum* B1-6 [170] and *Lactobacillus amylolyticus* L6 [59]. However, practical considerations such as enhancing product stability and establishing cost-effective manufacturing processes remain critical. In summary, while small-scale studies have illuminated the potential to valorize soybean byproducts, scaling up these processes to industrial levels presents challenges of maintaining consistency, efficiency, and cost-effectiveness.

### 9.7 Perishability and environmental impact

Soybean byproducts, particularly okara and whey, are highly perishable, with a short shelf life that leads to rapid degradation, risking both food waste and economic losses along the production chain [1,19,57]. To valorize these byproducts, methods like extrusion, drying, and fermentation are employed, but many of these are energy-intensive, raising production costs and environmental concerns. As such, energy-efficient approaches are critical to enhance both economic and ecological sustainability. For example, Voss, Monteiro, Jauregi, Valente and Pintado [171] developed a lactose-free prebiotic and symbiotic beverage by hydrolyzing fresh okara with *Cynara cardunculus* proteases, followed by fermentation with *Lactobacillus rhamnosus* R11 and/or *Bifidobacterium animalis* ssp. *lactis* Bb12. This approach not only boosted bioactive compound levels and enhanced isoflavone bioavailability but also improved angiotensin-converting enzyme inhibitory activity [172], beneficial for cardiovascular health, all while avoiding energy-intensive drying processes.

## 10 Conclusions

While the food industry inevitably generates waste and byproducts, current management practices often overlook their valuable potential, contributing to environmental challenges. Many valorization strategies focus on low-value applications like energy generation, whereas food, pharmaceutical, and bioplastic industries could benefit from utilizing these byproducts as cost-effective raw materials for high-value products. The soybean industry, in particular, produces diverse byproducts, including okara, soy whey, soy hulls, soy meal, and lecithin, all of which hold significant promise for sustainable valorization. To unlock this potential, the adoption of innovative solutions is essential, especially in overcoming challenges such as anti-nutrients, allergens, and issues related to concentration, drying, viscosity, and solubility. Integrated value chains that maximize byproduct utilization and establish closed-loop systems offer a path toward achieving zero-waste and improving sustainability within the industry. Drawing insights from successful models, such as those in dairy whey processing, can help overcome existing barriers and facilitate the transition to more efficient, environmentally friendly, and economically viable practices. To fully capitalize on the value of food byproducts, cost-effective methods that transform these materials into bioactive-rich food ingredients must be prioritized. However, the environmental impact of recovery technologies, particularly concerning solvents and chemicals, requires careful consideration. Simplifying processes and developing scalable, industry-suitable methods will be crucial for enhancing the applicability and relevance of byproduct valorization in the coming decades. Through collaboration across the supply chain and continued innovation, the soybean industry can transition towards a sustainable, circular bioeconomy, minimizing waste and maximizing value.

## CRediT authorship contribution statement

**Ahasanul Karim:** Writing – review & editing, Writing – original draft, Visualization, Conceptualization. **Emmanuel Freddy Osse:** Writing – review & editing. **Seddik Khalloufi:** Writing – review & editing, Visualization, Supervision, Funding acquisition, Conceptualization.

## Funding

This work was supported by the 10.13039/501100000038Natural Sciences and Engineering Research Council of Canada (NSERC) under grant number RDC 538873-19.

## Declaration of competing interest

The authors declare that they have no known competing financial interests or personal relationships that could have appeared to influence the work reported in this paper.
